# Watching the eye with Mars in sight

**DOI:** 10.1113/EP092974

**Published:** 2025-08-10

**Authors:** Peter zu Eulenburg, Lonnie Petersen, Damian M. Bailey

**Affiliations:** ^1^ Institute for Neuroradiology University Hospital Ludwig‐Maximilians‐University Munich Munich Germany; ^2^ Department of Biomedical Sciences, Faculty of Health Sciences University of Copenhagen Copenhagen Denmark; ^3^ Department of Aeronautics and Astronautics Massachusetts Institute of Technology Cambridge Massachusetts USA; ^4^ Institute for Medical Engineering and Science Massachusetts Institute of Technology Cambridge Massachusetts USA; ^5^ Neurovascular Research Laboratory, Faculty of Life Sciences and Education University of South Wales Pontypridd UK; ^6^ Bexorg, Inc. New Haven Connecticut USA

**Keywords:** brain, eye, microgravity, SANS, spaceflight

1

The initial terminology for the observed changes in visual acuity in astronauts during long‐duration spaceflight (LDSF) was visual impairment owing to intracranial pressure (VIIP), because all five symptomatic LDSF astronauts with postflight lumbar punctures had shown an elevated opening pressure even weeks after a landing. The National Aeronautics and Space Administration (NASA) then went on to rename VIIP as spaceflight‐associated neuro‐ocular syndrome (SANS) and, at the same time, refrained from using the word papilloedema in the publications, opting for the more neutral description optic disc oedema instead. In hindsight, from the perspective of European researchers, it remains unclear to us whether this was entirely motivated on clinical and scientific grounds or a mere marketing strategy to avoid scaring away political funding. A slowly developing hydrocephalus in microgravity certainly does not sound desirable to aspiring space cadets. But the rebranding from VIIP to SANS did more to obscure than directly to address this major human health risk, which is currently classified as ‘red risk’ by the agency. And in the long term, the aim of all involved researchers and physicians is to derisk human spaceflight and exploration FIGURE 1.

**FIGURE 1:**
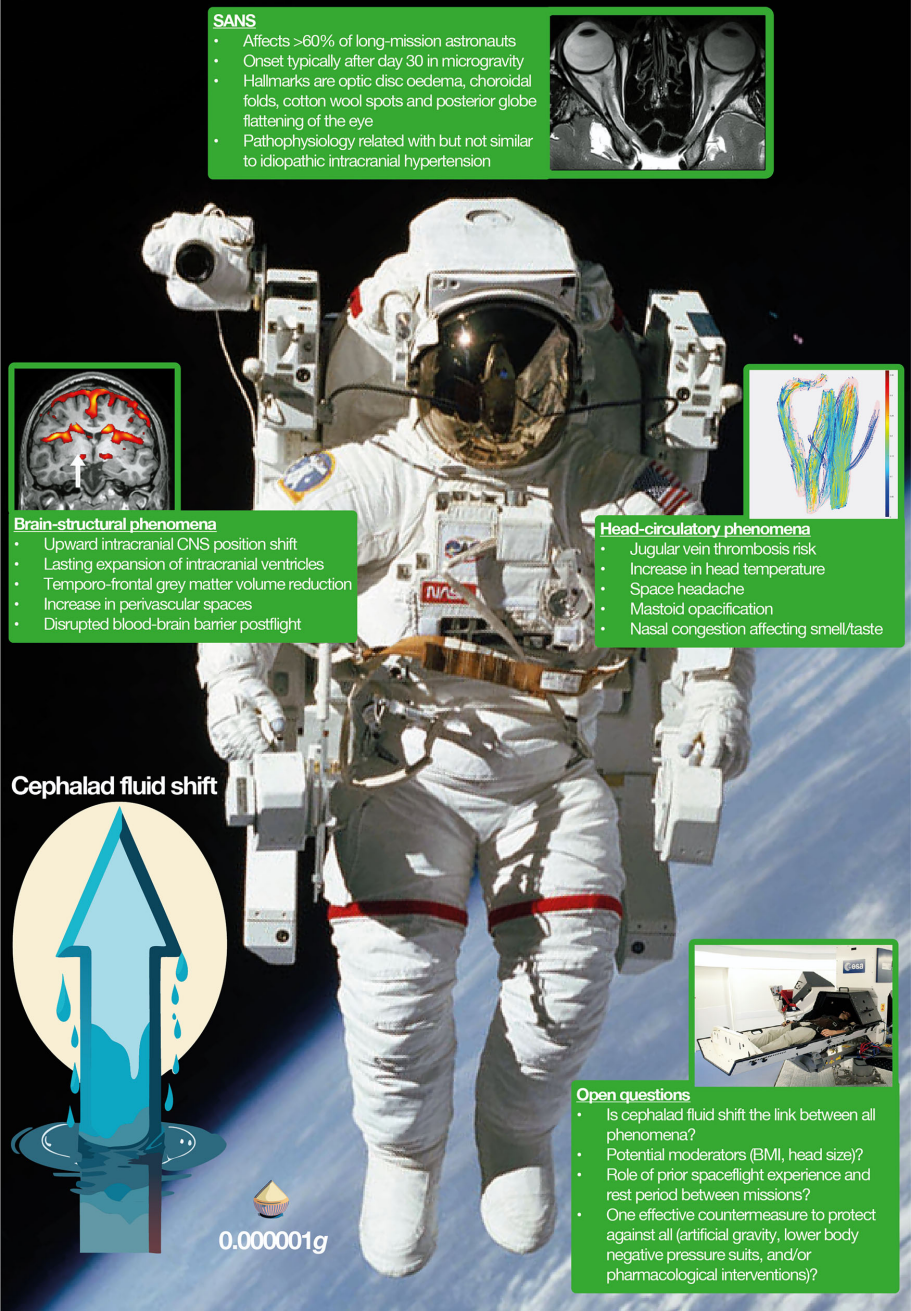
Overview of the main phenomena thought to be part of the cranial microgravity response in long‐duration spaceflight (>30 days). Spaceflight‐associated neuro‐ocular syndrome (top box) is the initial ocular finding, accompanied by the changes in brain structure (left box) and cranial circulatory response (right box). All these alterations are likely to be caused by the cephalad fluid shift in microgravity. Abbreviations: BMI, body mass index; CMR, cranial microgravity response; SANS, spaceflight‐associated neuro‐ocular syndrome.

In this issue of *Experimental Physiology*, Ng and Mollan (2026) provide a comprehensive review of SANS. The authors not only put SANS into a general physiological and clinical context but also highlight the translational benefits of terrestrial data that might help to inform the prevention and treatment of SANS (in space). SANS affects the eyeball structure, the optic nerve sheath and visual acuity in ≤70% of astronauts exposed to long‐term microgravity. Although the effects on visual acuity quickly resolve upon return to a ‘normal’ (i.e., 1 G) gravitational environment, structural damage to the eyeball (e.g., choroidal folds) can last for decades after a mission in space that lasts >60 days. The past decade of spaceflight research has underscored the crucial need to protect the eye and brain as prerequisites for safe and sustainable human travel beyond the Moon.

Can the eye (SANS, in particular) serve as a prototype condition for microgravity exposure, enabling simultaneous investigation of countermeasures to protect both the brain and the head? Or is SANS a unique entity and exclusively an ocular satellite phenomenon caused by long exposure to microgravity? We believe that the answer to the first question is ‘yes’ and to the second ‘no’. A holistic countermeasure strategy is needed to protect the entire CNS in space from developing multiple complications that include not only SANS but also others, such as hydrocephalus, elevated head temperature, space headache and the risk of jugular vein thrombosis. All these medical risks for astronauts are potentially unified by some level of venous and/or CSF outflow resistance subsequent to microgravity‐induced cephalad fluid shifts. Moreover, we suggest that altered compliance of cerebral structures might be an additional culprit.

To advance our understanding of SANS, integrative physiologists, flight surgeons, ophthalmologists, neurologists, neuro‐ and cognitive scientists should engage in more collaborative, cross‐agency research efforts, particularly to overcome the interpretive limitations posed by small cohort sizes. There is an urgent need for objective, quantifiable diagnostic and prognostic biomarkers, which will probably emerge from SANS research, with optical coherence tomography highlighted as a key modality in this review. By harmonizing and densely sampling optical coherence tomography parameters across international space agencies, it might finally become feasible to construct a dose–response relationship and, finally, create a reliable computational model to characterize the collective impact of microgravity exposure. Several questions remain unanswered: are two 90‐day missions similar to one 180‐day mission? What about two half‐year missions separated by a 2‐year recovery on Earth? The answers to these questions will have an effect on planning the 1000‐day return journey to Mars in the future.

The time point of onset for SANS in a first‐time flyer seems clearer. But the delayed onset and progression of SANS also means that we need bed rest studies longer than the typical 30–45 days used by present campaigns to understand this phenomenon fully. The European Space Agency has since begun to plan and conduct longer bed rest studies (also upon request of the partner space agencies). As eloquently highlighted in the review by Ng and Mollan (2026), bed rest studies using a 6° head‐down tilt for 30 days, although ethically permissible for healthy participants, do not fully replicate the spaceflight environment required to induce clinically relevant SANS. To simulate the ocular effects of long‐duration microgravity more accurately, future studies might need to adopt steeper tilt angles (e.g., 8°–10° head‐down tilt) combined with extended durations of bed rest. Only in these more physiologically demanding conditions can we meaningfully assess whether Earth‐based countermeasures offer true protective benefit during LDSF, including a mission to Mars.

SANS develops slowly once you arrive in orbit. If we apply the present threshold of a >20 µm increase in total retinal thickness, then one can typically detect the onset of SANS 30–60 days into a planned half‐year mission. It is therefore unlikely that space tourism and transfer missions to the Moon will be affected by SANS, as indeed was the case with the Space Shuttle and the Apollo missions. The delayed recognition of SANS can be attributed, in part, to the late structural onset of the condition, which became apparent only as LDSF missions became increasingly more common. Between 2005 and 2010, average mission durations on NASA and European Space Agency platforms increased significantly (from ∼10 to >100 days), marking a crucial shift in exposure to microgravity‐related physiological stressors. Before the construction of the International Space Station, only the final Skylab mission (84 days) approached similar durations, whereas Russian cosmonauts had already accumulated extensive experience on long‐duration missions (>60 days) aboard orbital stations such as MIR. In fact, Russian researchers were the first to document postflight papilloedema in 50% of their cosmonauts returning from MIR, suggesting that these cases (Myasnikov & Stepanova, 2008) might have been the earliest manifestations of what is now recognized as SANS.

Reviews and research articles on SANS often discuss the difference between intracranial pressure (ICP) in a supine or standing position as a valid reference point for comparison with the phenomenon observed in microgravity. A revized conceptual framing of ICP (mis)assumptions is warranted to account for the growing body of evidence documenting intracranial fluid shifts in LDSFs. These include optic nerve sheath expansion with associated optic disc oedema, facial puffiness, nasal congestion, ventricular enlargement suggestive of external hydrocephalus, mastoid opacification indicating fluid saturation, and increased cross‐sectional area and disturbed flow (including stagnation or reversal) within the internal jugular veins. These findings collectively challenge the sufficiency of current ICP‐centric models and highlight the need for a broader, more functionally integrated systems‐based understanding of cephalad fluid dynamics in microgravity.

The average ICP appears elevated in microgravity compared with Earth‐based conditions, where typical diurnal variation includes 7–9 h of sleep in the supine position and the remainder of the day spent in partial (sitting) or full upright postures. Notably, the rhesus monkey Krosh (the only primate to date implanted with a pressure sensor) demonstrated a sustained 25%–30 % elevation in ICP over the 12‐day *Bion* 10 mission relative to the preflight control baseline. In the 2022 SANS‐CM bed rest study on countermeasures, two daily 3 h periods of upright sitting ablated structural ocular changes indicative of SANS measured by optical coherence tomography (Brunstetter, 2023). These findings suggest that regular upright posture in a 1 G environment is essential for effective cranial fluid drainage and clearance of protein waste. In the absence of this, prolonged cephalad fluid shift in microgravity might lead to the accumulation of proteinaceous material and delayed glymphatic clearance, as evidenced by a >3‐week ‘amyloid washout’ phase observed via brain‐derived blood‐borne biomarkers in a recent pilot study (Zu Eulenburg et al., 2021).

The potential role of body mass index as a factor contributing to SANS is also addressed by Ng and Mollan (2026). We encourage future research to incorporate individual head size (quantified as total intracranial volume) in combination with body mass index as (SANS‐mediating) covariates. Together, these factors might account for a substantial portion of the inter‐individual variance observed in SANS‐related outcomes. Importantly, this approach could also reduce the reliance on dichotomous sex‐based comparisons in small‐sample studies, because total intracranial volume alone explains most structural intracranial differences typically attributed to biological sex.

All domains of human and non‐human space exploration research are flooded with acronyms. This can prove a memory hurdle for aspiring researchers. Nonetheless, we feel that there is still a term and acronym lacking to cover and embrace all known (patho‐)physiological consequences of cephalad fluid shifts to the head. At this point in time, SANS is currently drowning out all other domains of neuroscience and neurology literature represented during LDSF. And it seems mostly because the lion's share of funding has been thrown at the topic since 2011. But SANS is most likely to be only one of several consequences of cephalad fluid shift in microgravity. Hydrocephalus associated with LDSF (HALS) is another syndrome and most probably related, but rarely acknowledged (Roberts & Petersen, 2019). HALS was introduced to describe the expansion of intracranial CSF compartments and increased free water content across brain tissues. However, LDSF astronauts also experience a constellation of additional signs (anosmia owing to nasal congestion, mild headaches, mastoid opacification, elevated head temperature and increased risk of jugular vein thrombosis) all likely to be linked to cephalad fluid shift and impaired cranial venous outflow (Seidler et al., 2024). These phenomena, often associated with SANS, suggest the need for a broader conceptual framework. We propose the term cranial microgravity response (CMR) as a unifying physiological construct. The review by Ng and Mollan (2026) represents a key step towards establishing such an integrative perspective.

## AUTHOR CONTRIBUTIONS

Peter zu Eulenburg and Damian M. Bailey conceptualized the manuscript. PZE drafted the manuscript. All authors took part in the revision and editing process of the work. All authors approved the final version of the manuscript.

## CONFLICT OF INTEREST

Damian M. Bailey is Editor‐in‐Chief of *Experimental Physiology*, former Chair of the Life Sciences Working Group, former member of the Human Spaceflight and Exploration Science Advisory Committee to the European Space Agency, current member of the Space Exploration Advisory Committee to the UK and Swedish National Space Agencies and current member of the National Cardiovascular Network for Wales and South‐East Wales Vascular Network. Damian M. Bailey is also affiliated with Bexorg, Inc. (USA), focused on the technological development of new biomarkers of cerebral bioenergetic function and structural damage in humans. Lonnie Petersen is a current member of the Life Sciences Working Group to the European Space Agency (ESA) and lead scientist (Massachusetts Institute of Technology Principal Investigator) for the Translational Institute for Space Health, Human Research Program, NASA. Peter zu Eulenburg is a former member of the Life Sciences Working Group and a current member of the most senior advisory body, the Human Spaceflight and Exploration Science Advisory Committee (HESAC), to ESA.
